# CareSearch: finding and evaluating Australia's missing palliative care literature

**DOI:** 10.1186/1472-684X-4-4

**Published:** 2005-08-08

**Authors:** Jennifer J Tieman, Amy P Abernethy, Belinda S Fazekas, David C Currow

**Affiliations:** 1 Department of Palliative and Supportive Services, Flinders University, Adelaide South Australia, Australia; 2 Southern Adelaide Palliative Services, Repatriation General Hospital, Daw Park, South Australia, Australia; 3 Division of Medical Oncology, Department of Medicine, Duke University Medical Centre, Durham, North Carolina, USA

## Abstract

**Background:**

Palliative care is an evolving specialty with a growing evidence base. However, evidence is less accessible than it could be with a lower than average conversion of conference abstracts to articles in peer-reviewed journals and the need for more accessible tools to support evidence-based practice (EBP) in palliative care. The CareSearch project involved identifying, collecting and evaluating Australia's "grey" palliative care literature and identifying international published literature missing from the electronic indexing systems. The literature was then catalogued and made publicly available through the CareSearch website.

**Results:**

To date over 2,500 items have been included in the CareSearch database and can be accessed and searched through a publicly available website. Nearly 2,000 items are conference abstracts and 178 are theses or government, organisational and planning documents. A further 410 items relate to articles from palliative journals that are not indexed on a major bibliographic database. The website also provides tools and facilities to support palliative care practice and research.

**Conclusion:**

CareSearch is a new evidence resource for palliative practitioners, educators and researchers. The palliative community now has access to a more comprehensive literature base as well as a resource that supports the integration of knowledge into practice. This specialised data repository enables users to access information on the body of work that has shaped palliative care development and prevents the potential loss or duplication of research work. It also provides a template for other emerging disciplines to use in capturing their literature and evidence.

## Background

Palliative care has only been a distinct academic discipline in Australia since the 1980s, with an emerging publication base. Defining the work already done that contributes to evidence-based practice and research directions is time consuming and difficult. This problem is shared with many clinical disciplines especially those that are relatively young such as rehabilitation, geriatric medicine, sports medicine and sexual health.

The role of evidence in the practice of health care is built on a number of concepts relating to structured use of information, an evaluation of material and the use of best available evidence. Sackett and colleagues (1996) define evidence-based practice as follows:

*Evidence based medicine is the conscientious, explicit, and judicious use of current best evidence in making decisions about the care of individual patients. The practice of evidence based medicine means integrating individual clinical expertise with the best available external clinical evidence from systematic research (P71) *[1].

As an emerging field, the early literature was not always captured within the peer-reviewed literature and is still difficult to access. There is a perception among researchers, clinicians and funders that the amount of information available in an accessible form does not reflect the research and planning that has occurred. Poor accessibility is worsened by:

• Presentation of work at conferences without preparation of a subsequent peer reviewed manuscript;

• Researchers who do not communicate their work in any forum;

• The breadth of clinical scope for good palliative care [2];

• Slow emergence of specific peer-reviewed publications as a new discipline develops;

• Inconsistent cataloguing of the palliative care literature in major electronic bibliographic databases; and,

• Difficulties in searching for palliative care literature due to sub-optimal search strategies.

These problems make the palliative care evidence pool appear less comprehensive than it is.

For example, the journal *Palliative Medicine *began in 1987 but was not indexed in MEDLINE or EMBASE until 1992; *Death Studies *was indexed in MEDLINE until 1980 and then not reliably indexed again until 1997 while it was variably indexed in EMBASE from 1984 onwards. After indexing on electronic bibliographic databases, the variability in indexing can be seen when citations accessed in major electronic bibliographies are compared with hand searches [3,4].

For a field such as palliative care, issues around evidence are made more difficult by the multidisciplinary and multispecialty nature of the clinical care. The list of relevant journal resources spans all specialties and sub-specialties, just as prognostication and care of patients with life-limiting illness span all diseases. Limiting the palliative care resources to those published in palliative medicine sub-specialty journals severely limits the breadth of information available to inform best practice. Articles relevant to palliative care can be found in sources from gastroenterology, respiratory, cardiology, surgery, nursing and paediatrics specialty journals to the health education, health promotion and occupational therapy literature. Relevant information can also be found in the literature of non-bioclinical disciplines such as pastoral care and social work. This creates additional hurdles in locating and evaluating pertinent literature [5].

Grey literature is defined as "that which is produced on all levels of government, academics, business and industry in print and electronic forms but which is not controlled by commercial publishers" [6]. It includes materials such as dissertations, census and statistical data, reports of research (completed and uncompleted), and technical reports. It does not mean that grey literature is not peer reviewed and indeed it may have undergone extensive review. Citations of these works are usually left out of the major bibliographic databases.

This "missing" literature and grey literature is particularly significant in emerging fields such as palliative care where government-funded scoping studies, organisation-sponsored reports and other commissioned work often most accurately reflect the evolving state of current knowledge [5,7,8].

The complications of searching the palliative care literature – including incomplete indexing, multiple disciplines, and the grey literature – are amplified by the problems inherent to searching in any subject area. Reliance on a single electronic bibliographic database can reduce the ability to identify relevant articles. Various studies have shown that it is important to identify and search the bibliographic databases most relevant to the topic to improve the recall of target studies [9,10].

Electronic searching alone also reduces the results attained. Savoie and colleagues [11] demonstrated that extended searching beyond the major databases improved the identification of randomised controlled trials when compared to bibliographic index searching alone. Searching specialised databases like CareSearch and trial registers were the most important additional strategies after searching electronic bibliographic databases.

In order to be identified through any search, a basic requirement is that the author must have published the work in some forum. A recent review of studies dealing with acceptance rates and subsequent publication rates for biomedical abstracts found a conversion rate, from presentation to publication in full, of around 45% [12]. There is the perception that many researchers in the discipline of palliative care have presented their work at conferences, but not subsequently submitted manuscripts for peer-reviewed publication. Preliminary estimates from a review of CareSearch abstracts suggest a conversion rate of less than 20%. This problem is not confined to palliative care; the National Institutes of Health (NIH) found that only 80% of the studies they funded were published [13].

While the lack of publication may reflect that many presentations were not based on rigorous studies and hence did not meet publication criteria, for an evolving field the loss of conceptual work or preliminary studies remains a significant loss if it fails to inform the development of future work. Further, at times the best evidence will be grey literature as it is the only evidence that exists.

Publication bias, whether institutionalised or self-regulated by authors, has significant effects on the assessment of interventions. Exclusion of this literature (i.e. studies that are not published, have a limited distribution or are not included in bibliographic retrieval systems) can lead to a significant overestimation of an intervention's effect [14,15].

Systematic reviews can only claim to be complete if the process involves systematic searching of published data including conference abstracts [11]. The savings of having one source for searching for these items are manifest, if such a collection continues to be updated.

In an effort to address some of these problems outlined above as they relate to palliative care, the Rural Health and Palliative Care Branch of the Australian Department of Health and Ageing provided $580 000 over 5 years from the National Palliative Care Program to fund the Evidence Based (CareSearch) Project. Flinders University was contracted by the Australian Government to undertake the work. The University's Department of Palliative and Supportive Services has managed and executed the project.

The project has three specific aims:

• To capture and collate Australia's "missing" palliative care evidence and the international "missing" published literature in palliative care;

• To make this literature accessible to inform best practice; and

• To promote evidence-based practice (EBP) in palliative care through an electronic cyber-community.

The project is commonly called "CareSearch", reflecting its virtual home at the CareSearch website [16] and its focus on identification of literature relevant to palliative care.

## Implementation

### Project management

A National Reference Group of thirteen clinicians and researchers volunteer their time to oversee the project. They contribute their substantial skills in EBP, palliative care, general clinical care, information systems and research evaluation. The National Reference Group functions as an Editorial Board meeting twice a year to advise on directions and policy issues. The Project Team translates these broad directions into operational activities. The Project Team comprises the three part-time project staff members who carry out the work and the two investigators who act as a local resource and as advisors. The National Reference Group also assists by evaluating conference abstracts and by providing reviews for the "Hot Picks" section.

### Project parameters

The National Reference Group has provided advice on the issues and processes around the inclusion and assessment of materials selected for the literature databases. The considerations around the inclusion process recognised that often there is no randomised controlled trial or "gold standard" in the literature to address the clinical questions in palliative care and that clinicians may need to consider the "evidence pyramid" and look for the best available evidence. For some areas in palliative care there may not be any "formal" evidence available to support clinical judgement and as is the case with other areas of clincial practice, even may not relate to "this" patient. Evidence issues are compounded by the multidisciplinary nature of palliative care that draws on different methodological approaches and research paradigms. As a result the primary emphasis of the project was on identifying sources of information, evidence and commentary that could add to an evidence base given the broadest definition of "best available evidence".

In conjunction with the CareSearch Project Team, the National Reference Group identifies literature sources and developed a schema to organise and evaluate the collected materials and data. Information sources include those not available through the major electronic bibliographies such as abstracts from conference proceedings; reports and other literature from federal, state, and territory government departments and from palliative organisations; theses and treatises; and missing published literature representing non-indexed articles from palliative care journals (before the date of first citation and unpredictable omissions since that date). Collection and evaluation of material is an ongoing function of the project.

### Abstracts and conference proceedings

The National Reference Group identifies conferences held in Australia since 1980 that may include issues relevant to palliative care. The sponsoring organisation for each conference is contacted and permission sought to collect a copy of the conference proceedings or abstract book, copy pertinent abstracts, and add them to the CareSearch library. Once the abstract books are obtained, two National Reference Group members separately review each book and select relevant abstracts about the care of people with advanced life-limiting illness regardless of diagnosis. CareSearch continues to collect and evaluate new conference abstracts from each relevant conference or scientific meeting presented around Australia.

For level of evidence evaluation, a purpose driven proforma was developed [See additional file 1]. Using the proforma, evaluators record information about the study, identify key words, and decide whether data are presented in the abstract. The evaluation schema also looks at study question, study design, validity of the conclusions and generalisability of the results to clinical practice. For many abstracts the conference abstract evaluation is limited due to the limited information provided in the abstract. Evaluators must also exercise judgement with regard to indicating what could be the best study design to address the topic being covered by the conference presentation. However, this schema provides broad guidance for users regarding the quality of the work reported in the abstract and hence provides a caution about its possible application in practice.

Two reviewers independently rate each abstract and give it a final A, B or C classification. If the reviewers do not agree on the classification, a blinded third reviewer rates the abstract and the final classification is that which receives two of three nominations. The abstract and reviews are stored in the CareSearch database. If an author disagrees with the final classification, he or she can contact CareSearch directly to resolve the issue; the classification information will be removed from the website and the abstract will be sent out to a new pair of National Reference Group members for review.

### Reports & treatises

Listings of state, territory and federal departments with responsibility for palliative care activities were prepared together with a listing of national organisations with palliative interests and universities within Australia. All of these contacts have been approached in writing with follow-up phonecall for contributions to the CareSearch database. They have been informed of the nature of the project and permission has been obtained to provide the document abstracts or executive summaries on the CareSearch website. These organisations nominate documents for inclusion in CareSearch and the National Reference Group then reviews these selections for relevance. These documents are not further evaluated.

Academic institutions offering higher degrees in areas associated with palliative care were identified. The project then contacted the academic institution to nominate any Masters or PhD theses or academic treatises that are palliative in content. The nominated items are reviewed by the National Reference Group for relevance. The documents are entered into the literature database without further evaluation.

### Non-indexed journal articles

The National Reference Group formed a consensus opinion on twelve key journals for palliative care from an initial list of 51 journals seen to be relevant to palliative care. The journals were handsearched from the first published volume until July 2002 to identify all articles describing original research or significant reviews in the field. Ovid MEDLINE, EMBASE, CINAHL and PsycINFO were then searched for all of the identified articles. Reviews since July 2002 are ongoing. The "missing" published palliative care literature is defined as those articles published (as evidenced by handsearching) but not indexed in the major biomedical bibliographic databases. The publishers of the palliative care journals have been contacted, and if they agree, abstracts for the "missing" articles are added to the CareSearch library. A listing of the palliative care journals and those who agree to have their abstracts included is available on the CareSearch website.

### Database and website

A structured database houses all CareSearch data with the results available on a user-friendly public-access website. The website ensures systematic delivery of the key outcomes for all people who need to access this work. The database was built using Microsoft Access 2000 (Microsoft Corporation, Seattle, Washington, USA) and an interactive website was designed using Microsoft Visual Basic .NET and Microsoft SQL Server 2000. The website can be easily updated by project administrative staff using a content management system. The website layout and database access is being improved based on comments solicited from members of the National Reference Group and website users.

The primary function of the website is to house the CareSearch library and permit user access. The search engine allows unrestricted searches by any word (not limited to key words) and specific searches by author, database (e.g. conference abstract) or year. Boolean logic is used to refine searches.

## Results

### Abstracts and conference proceedings

To create the conference abstracts database, 25 conference organisations were approached in the initial investigation. All organisations supplied books. One hundred and eleven books of conference proceedings were reviewed and 1,690 conference abstracts were assessed as being relevant to palliative care. Additional abstracts have been added as part of the annual update cycle and as other relevant conferences are identified. Over 20% of these conference abstracts to date have been formally evaluated.

### Other literature

To date, 100 government and organisation sponsored documents have been located and included in the database. In addition, 78 theses from 14 Australian universities have been catalogued.

### Non-indexed articles

Twelve palliative care journals were reviewed from initial publication to July 2002. These journals were:

• Palliative Medicine

• American Journal of Hospice and Palliative Care

• Journal of Pain and Symptom Management

• Journal of Palliative Medicine

• Journal of Palliative Care

• International Journal of Palliative Nursing

• Progress in Palliative Care

• European Journal of Palliative Care

• Death Studies

• Supportive Care in Cancer

• Hospice Journal

• Psycho-Oncology

A total of 8,398 items were identified, with 7,557 (90%) indexed in one of the four main bibliographic databases. Of the 841 non-indexed items, 410 (49%) have been identified as research or commentary and been included in the CareSearch database. The other 431 items were not included as they were not deemed to be articles. They comprised excluded items such as book reviews, video reviews or conference summaries.

### Other evidence based resources

Based upon feedback from National Reference Group members and users, CareSearch is developing and updating multiple resources that support and foster EBP in palliative care. Current resources include a monthly "Hot Pick" literature review of a recent publication, a research resource area including a specialised platform to promote data management in multi-site clinical studies, palliative care audit tools, and a "search strategy generator" that facilitates efficient MEDLINE searching and generates up to date resources saving the website administrator the need to constantly review the currency of materials.

Members of the National Reference Group have agreed to provide reviews for the Hot Picks. They select an article from the literature that they believe is significant to the practice of palliative care and provide a written review highlighting its relevance.

There are currently eleven searches on palliative care topics available on the website. A further ten will be written in the coming year. Additional pages for the Services Models and Clinical Practices section will also be added in the coming year.

The CareSearch Research Platform has been developed to support research work within palliative care by providing access to a tool that

• enables the online design of data collection forms and questionnaires;

• allows for web-based and email-based form completion through the CareSearch website;

• enables data entry from multiple sites with a single co-ordinating research site;

• provides for basic reporting of results with features such as percentages, graphs, and tables; and

• allows export of data to other programs such as Excel, Access or SPSS.

With a user-friendly interface, the tool encourages beginning researchers and small services who may not have access to statistical and research resources as well as supporting larger multi-site research activities. The capacity to webhost the studies to allow online data entry from multiple sites is particularly beneficial to agencies that do not have access to such resources. This platform has already been used to collect data for several projects including an international multisite clinical trial. Given specific challenges in participant accrual in palliative studies a tool available internationally for multisite research is critical to generate better quality evidence in clinical areas.

The project has only just begun to look at the issues associated with the development of online communities that could play a significant role in knowledge dissemination and translation. New project directions are being informed by the developing body of literature relating to communities of practice and knowledge translation [17-20]. Bulletin boards and targeted resources and tools for special interest groups will be introduced in the coming year.

### Database and website

In total over 2,500 items that are missing from the formally indexed palliative care literature have already been located, evaluated, catalogued and combined into the CareSearch database. This continues to grow. Access to the database items is through the CareSearch website [16]. There has been consistent expansion in the use of the website since it became available in March 2004 (See Table 1). The CareSearch website tracks usage through the LiveSTATS.XSP log analyser (LiveStats Service Provider Edition 6.2). While care needs to be exercised in the interpretation of website statistics, they provide a useful indicator of site usage. The Visits report shows the number of visitor sessions to CareSearch during a specified period. A visit refers to a series of requests from a uniquely identified client. Website statistics show a three-fold increase in the number of visits to the site in the eleven months to January 2005, with a high level of repeat use, suggesting that users find the material relevant and useful. The increasing number of distinct internet service providers (ISPs) and companies visiting the site suggests that new users are able to locate the website.

**Table 1:** Visitor statistics: CareSearch from March 2004 – January 2005

**Month**	**Total visits**	**Distinct ISPs or companies visiting the site**
March 04	413	77
April 04	360	90
May 04	636	144
June 04	925	153
July 04	1047	173
August 04	1137	216
September 04	1190	252
October 04	868	221
November 04	1065	302
December 04	1381	265
January 05	2051	275

## Discussion

The volume of data and materials identified supports the initial perception that there was a large "missing" literature not previously collated. CareSearch provides access to this "missing" non-indexed palliative care literature, complements the existing bibliographic databases and extends the coverage of palliative materials. Literature formerly only available through extensive handsearching, which could be of great significance in systematic reviews and meta-analyses, is now electronically available [21]. Over 2,500 items are stored in the CareSearch library. Inadequate access to this "missing" literature allowed a knowledge deficit and left the research community at risk of repeating completed work rather than building on existing knowledge. CareSearch is a critical platform for rectifying these problems, facilitating EBP and highlighting future research directions. The website's structure and features support EBP and the development of critical skills for identification and appraisal of information through access to search strategies, the inclusion of evidence hierarchies, audit tools and current literature summaries.

The palliative care community now has access to a more comprehensive literature base as well as a resource that supports integration of knowledge into practice. As much of the collected material falls within the concept of "grey literature" it may not have been subject to the same peer-reviewed process as published literature and may therefore be more variable in terms of evidence standards. However, this material captures not only the developmental history of the discipline, but also some of the knowledge that is missing from the formal peer-reviewed bibliographic databases for many different reasons and which can add value to future research and current practice.

For the discipline itself, CareSearch has systematically demonstrated that there is an Australian evidence base contributing to palliative practice and highlights this output in a public forum. The project has also provided a template and model for other emerging disciplines to capture their research and commentary to support the progress of the field.

The CareSearch approach to comprehensive coverage of the Australian palliative care information also facilitates identification of gaps in knowledge. This can promote thoughtful research questions and more appropriate use of existing data to support developments in clinical research including data for new studies' power calculations and recruitment, clinical care, service planning, funding and practice change. There is also the capacity to build linkages between researchers and encourage collaborative Australian projects building on existing local data.

The non-indexed published literature portion of the library reflects worldwide input. As the CareSearch team seeks to expand coverage more widely outside of the Pacific region, it will need to partner with other organisations and interested parties to build an understanding of the total state of current knowledge in palliative care including gaps and future directions.

The role of online literature and evidence in supporting clinical practice and research within Australia has been highlighted in the recent evaluation study of the Clinical Improvement Access Program [22]. This study found patterns of use of online resources that increased with patient admissions. This pattern coupled with self-report of use by clinicians showed that health professionals were using online evidence to support clinical decision-making. Clinicians, educators, researchers and healthcare planners in palliative care now have similar benefits through access to assembled and evaluated clinical and service resources.

Unpublished abstracts and other literature contribute to the knowledge base and need to be considered within the wider context of evidence. Randomised controlled trials are rare in palliative care and it is important to keep in mind that best evidence is the best established information available to answer the question at hand [1,23]. To date, less rigorous studies have figured more prominently in palliative care decision making than in other disciplines because that is what is available. Highlighting the current state of evidence through projects like CareSearch may support the arguments for further research [5].

To support continuing clinical and practice improvements, it is important that the existing databases retain their currency. The databases must continue to capture the new evidence and material as they are released to ensure that CareSearch is a "living" anthology. The website will also need to expand to incorporate the identified and emerging needs of the palliative care community with regard to information and evidence. The National Reference Group and the local CareSearch Project Team have already identified a number of future directions, including:

• Exploring mechanisms to evaluate and refer to websites themselves given their increasing influence as an information source;

• Increasing the reach of the project by actively engaging the wider palliative community with the contents and philosophy of the CareSearch website;

• Including abstracts from palliative related journals that are not indexed in the common electronic bibliographic systems;

• Building the critical appraisal capacity of the palliative care community by widening participation in the evaluation of conference abstracts;

• Validating search filters to support effective searching for palliative care information within the general medical and health literature;

• Developing descriptors to enable generalisability of results across palliative patient populations;

• Investigating the use of the website as a benchmarking and audit service for palliative care services;

• Increasing the usefulness of the Research platforms by creating template surveys and including formatted tools for use in research;

• Partnering with palliative care communities around the globe to foster evidence-based palliative care internationally; and

• Using CareSearch as a template for other disciplines to develop their research and evidence databases.

For other groups who may be interested in using this approach to consolidate the knowledge and research materials for their discipline, it is important to be realistic about the time and efforts required to source and assess the materials. This type of project relies heavily on the goodwill and expertise of those involved in a volunteer capacity. It also requires substantial expertise and management skills to develop not only the internal processes but also carry out the negotiations with institutions, agencies and technology partners and meet the various legislative requirements including copyright, privacy and intellectual property.

## Conclusion

The palliative care literature readily available through the bibliographic indexing system may be less comprehensive than the actual pool of research, review and opinion. By identifying possible missing data and reviewing these materials, the Evidence-Based (CareSearch) Project has brought together not only Australia's palliative care literature but also key concepts relating to palliative care practice, planning and research. By embedding access to missing evidence resources within a user-friendly accessible structured framework, the CareSearch website actively promotes contemporary EBP within the palliative care community.

## Availability and requirements

• **Project name: **Evidence based (CareSearch) Project

• **Project home page: **

• **Operating system(s): **Windows 2003

• **Programming language: **MS VB .Net, MS C#, ASP

• **Other requirements: **Client – Internet Explorer 5.5 or later, Server – Minigen Content Manager

• **License: **Not Applicable

• **Any restrictions to use by non-academics: **None except for the Research Platform which requires registration with the CareSearch project

## Competing interests

The author(s) declare that they have no competing interests.

## Authors' contributions

DC and AA are co-investigators for the project. DC, AA, JT and BF were involved in the location and evaluation of the materials for inclusion and in the development of the websites and its resources. All authors have had intellectual and editorial input to this manuscript.

## Pre-publication history

The pre-publication history for this paper can be accessed here:



## Supplementary Material

Additional File 1 Conference Abstract Evaluation. Additional file 1 contains the schedule used by reviewers for the evaluation of conference abstracts entered into the CareSearch database. It also details the instructions and procedures for abstract evaluators and lists the review areas for the conference abstract evaluation.
Click here for file
