# Supplementary material for: CareSearch: finding and evaluating Australia's missing palliative care literature
Source: BMC Palliat Care. 2005 Aug 8;4:4. doi: 10.1186/1472-684X-4-4 (PMC1190188; doi:10.1186/1472-684X-4-4)
Supplement: Additional File 1 — Conference Abstract Evaluation. Additional file 1 contains the schedule used by reviewers for the evaluation of conference abstracts entered into the CareSearch database. It also details the instructions and procedures for abstract evaluators and lists the review areas for the conference abstract evaluation. [file 1472-684X-4-4-S1.doc]

# Additional file 1 – Conference Abstract Evaluation

### Evaluation schedule: Conference abstracts

**Cool Star**

Y/N

**Minimum Descriptive Information**: (Group C)

**Number:**

Y/N

**Indigenous**

**Enough information to determine further**

Yes

No

**categorisation?**

*(i.e. no data, review, descriptive, plenary or workshop)*

***Is there an associated Journal Publication*** *(searching by first/last author)***?:**

Yes

Unknown

No

_____________________________________________________________________

**Complete System Information**: (Group B)

**Keywords/Subject Headings;** Discipline: *(optional)* _______________

Primary Keyword: _______________

Keywords: _______________

_______________

_______________

**Question Being Asked?** (*text*) ____________________________________________

_____________________________________________________________________

**Study Category** *(see list)* _____________________

**What is the best study design to answer this question?**  ____________________

**What is this study design or type?** *(see list)* ____________________

**Interpretation**: **(**Group A)

**Relevance**

or

1 Very relevant

2 Relevant

3 Of some relevance

4 Of little or no relevance

5 Cannot Comment

**Scientific Merit**

or

1 Demonstrates excellent scientific merit and rigour

2 Demonstrates good scientific merit and rigour

3 Fair level of scientific merit and rigour

4 Poor level of scientific merit and rigour

5 Cannot Comment

**Applicability / Transferability**

or

1 Very applicable /transferable

2 Applicable /transferable

3 Somewhat Applicable / Transferable

4 Not

5 Cannot Comment

**Comments:** (*text*) ______________________________________________________

__________________________________________________________________________________________________________________________________________

**Evaluators**

**Final Group**

# CareSearch: evaluation of conference abstracts

# Procedure and instructions for evaluators

**Background**

CareSearch is a website that provides clinicians, researchers and educators in palliative care with access to sources of information to complement those readily available on the electronic literature databases, strategies to assist them in searching those electronic databases and a mechanism to support multisite research in this field.

The literature database found on CareSearch comprises four individual libraries – articles from 12 journals that have not been indexed and included on the main electronic databases such as Medline; conference abstracts from Australia; Australian treatises and theses; and grey literature from within Australia.

The conference citations and abstracts are selected from conferences held within Australasia from 1980. Each book of proceedings was searched for palliative care relevance, and then evaluated for level of evidence and quality. The conference abstract library within the Caresearch database provides the citation information, the abstract, the conference details and the evaluation.

**Evaluation of Conference Abstracts**

The evaluation of the conference abstracts is carried out online through the CareSearch website. Two evaluators who are clinicians or researchers working within the palliative field evaluate conference abstracts. The evaluation process uses a standard proforma.

Each evaluator nominates a set of review areas within which they are willing to evaluate abstracts. (See Attachment A) The evaluator will be sent a password enabling them to access the evaluation system.

The Project Manager initially assigns the review area to the individual conference abstract. The Website Administrator then assigns individual abstracts to evaluators on-line. This generates an email advising the evaluator that they have a new conference abstract to evaluate.

The evaluator will then enter the site and complete the on-line forms. Help is available in the side bar describing the functions and purposes.

In determining a grouping for the conference abstract, the system will automatically assign a “C” if the evaluator indicates that there is insufficient information to determine further categorisation. After completing the section dealing with content and study methodology, if the evaluator indicates that further evaluation is possible, the system will automatically assign a “B”. An evaluator is able to modify this classification to “A”, “B” or “C”.

A "C" grouping reflects work in progress (such as a conference report on a study that is planned or in the data collection phase), workshops or training sessions, or plenaries and topic overviews. It may also reflect a study whose study design was inappropriate or whose data analysis was flawed.

A "B" grouping indicates the benchmark standard for research or clinical studies where the question, study approach and analysis are all appropriate.

An "A" grouping is given when the abstract describes high calibre work dealing with a significant question for the field.

The "Cool Star" is an alternative/additional categorisation that highlights research or thought that is ground breaking or concept shifting. It does not necessarily relate to the study design and is applied independently of the grouping. So for instance a plenary that deals with an innovative perspective may be grouped as a "C" but could receive a "Cool Star". Alternatively a new methodological approach to answering a clinical or research question may gain a "Cool Star".

The grouping assigned by the evaluator will be uploaded to the website at the completion of the evaluation. Where the two evaluator grade the abstract the same, that classification will show up on the abstract’s citation details. If the two evaluations do not match, the Website Administrator will be automatically notified. A third evaluator will be assigned.

**Evaluator Feedback**

Feedback from evaluators is useful. Firstly, evaluators can advise the Project team whether they believe a conference abstract should not be included on the website, as it is not palliative in content. Evaluators can also assist by indicating if they are aware of a publication resulting from or associated with the abstract they are reviewing. Finally evaluators can indicate how the evaluation process could be improved or could be made more useful to the palliative community.

# Evaluation of conference abstracts: review areas

Pilot Studies

Qualitative Studies

Plenary Reviews/workshops

Intervention/Case Studies

Epidemiology

Retrospective Study (Intervention)

Quality Improvement/Quality Assurance

Complementary Therapy

Education

Costings

Policy (Agendas)/Service Models

Descriptive/Review/Literature Review/Case Series
